# In vitro angiogenesis and expression of nuclear factor κB and VEGF in high and low metastasis cell lines of salivary gland Adenoid Cystic Carcinoma

**DOI:** 10.1186/1471-2407-7-95

**Published:** 2007-06-01

**Authors:** Jiali Zhang, Bin Peng

**Affiliations:** 1 Key Lab for Oral Biomedical Engineering of Ministry of Education, School and Hospital of Stomatology, Wuhan University, China

## Abstract

**Background:**

Adenoid cystic carcinoma is a high malignant carcinoma characterized by intensive local invasion and high incidence of distant metastasis. Although many reports have demonstrated that angiogenesis has played an important role in tumor metastasis, the relationship between metastasis characters and angiogenesis ability in high and low metastasis cell lines of Adenoid cystic carcinoma has rarely been reported. The present study aimed to compare the angiogenesis ability of ACC-M (high metastasis) and ACC-2 (low metastasis) cell lines in vitro. Furthermore, the activity of nuclear factor κappa B and the expression of vascular endothelial growth factor (VEGF) in ACC-2 and ACC-M were also detected.

**Methods:**

Electrophoretic mobility shift assay was used to detect nuclear factor κappa B activity. Semi-quantitative RT-PCR was used to quantify the mRNA level of VEGF. Immuofluorescence double staining and semi-quantitative confocal laser scanning analysis was carried out to detect nuclear factor κappa B nuclear localization and staining intensity of VEGF. The angiogenesis ability of ACC-M and ACC-2 was compared by an in vitro three-dimensional angiogenic model assay. The vector transfection assay was performed to transfect the PCMV-IκBαM vector into ACCs cell lines expressing the phosphorylation defective IκBαM.

**Results:**

Nuclear factor κappa B activity and the rate of nuclear factor κappa B nuclear localization in ACC-M was significantly higher than that in ACC-2. Moreover, ACC-M exhibited higher mRNA and protein levels of vascular endothelial growth factor than ACC-2. VEGF mRNA expression was effectively decreased by inhibition of nuclear factor κappa B activity. Furthermore, ACC-M could remarkably stimulate the migration and tube formation of endothelial cells and induce The umbilical vein endothelial cells sprouting into the gel matrix.

**Conclusion:**

These results implicated that ACCs cells with higher metastasis feature might present greater angiogenesis ability.

## Introduction

Adenoid cystic carcinoma of salivary glands (ACCs) is a high malignant carcinoma characterized by intensive local invasion and insidious distant metastasis to the lung at an early stage, which is responsible for a poor long-term survival rate [1]. Although reasons of the invasiveness and aggressive metastatic dissemination of ACCs remain unclear, angiogenesis might be a possible involving mechanism [2]. Our previous study showed that the ACCs histological type which presented high metastasis tendency exhibited higher microvessel density levels and more intensive expression of angiogenic related factors [3].

Angiogenesis, the development of new blood vessels from the pre-existing vascular beds, is an essential pathophysiologic event occurring in tumor growth and metastasis. There are various factors involved in angiogenesis, including the vascular endothelial growth factor (VEGF), which plays a key role in regulating tumor vascularization [4]. In addition, VEGF has demonstrated a major association with initiating the process of angiogenesis through regulating proliferation, migration, and differentiation of endothelial cells [5]. On the other hand, the nuclear transcription factor κB (NF-κB) that was reported increased in cancers and promoted tumor angiogenesis [6]. Once activated, NF-κB p50 and p65 will translocate into nuclear and up-regulates a number of genes necessary for the angiogenesis of tumors, which have κB binding sites in their promoter regions [7]. Further, recent evidence has indicated that an over-expression of NF-κB is the key components of the angiogenic cascade, which contribute to VEGF-induced angiogenesis through up-regulation of VEGF mRNA expression in many tumors [5,8]. Our previous study has demonstrated the expressed relationship between VEGF and NF-κB: the staining intensity of VEGF was significantly correlated with NF-κB nuclear localization rate in 80 ACCs clinical samples [3].

To our knowledge, however, there were few reports about the relationship between the distant metastasis character and the angiogenesis ability in ACCs, mainly because it is uncommon, and more than a decade of observation might be required to appreciate the prolonged clinical course in some patients. As a result, using an in vitro model to study the angiogenesis ability in high and low metastasis ACCs cell lines is necessary. The low metastasis cell line ACC-2 was established from ACCs in 1988 [9]**, **and the high metastasis cell line ACC-M was a highly metastastic clone to the lung selected from ACC-2 [10]. The metastatic rate was 96% vs. 18% for ACC-M and ACC-2 cell line. Since the high and low metastasis cell lines were established, reports have been made to explore the difference between the two cell lines on the cell and molecular level associated with tumor metastasis [11].

In our present research, the relationship between angiogenesis and metastasis in ACC-M and ACC-2 cell lines has been studied. We use the in vitro angiogenesis model to compare the angiogenesis abilities in high and low metastasis cell lines of human ACCs. Furthermore, the NF-κBp65 activity and VEGF expression levels in the two cell lines were also detected.

## Methods

### Cell culture

The high and low metastasis cell lines of human ACCs (ACC-2 and ACC-M) [12] were obtained from the China Center for Type Culture Collection. The umbilical vein endothelial cell line (UVEC) was from the Key Lab for Oral Biomedical Engineering of Ministry of Education at Wuhan University. Cells were maintained at 37°C in DMEM and supplemented with 10% fetal bovine serum (GIBCO, Trace Biosciences Ltd., Sydney, Australia) under 5% CO2/95% air atmosphere and passed at a 4–7 split before use in the following experiments.

To obtain the conditioned medium (CM), sub-confluent ACC-M and ACC-2 cells were serum starved for 12 h in 6 well plates (Greiner, Labotechnik, Germany). The medium was replaced with 1.0 ml serum-free DMEM medium, and the cells were then incubated for 12 h, after which the CM were obtained, stored at -20°C, and used for the following experiments. The viability of cells was estimated by CCK-8 kit (Japan, Kumamoto, Dojindo).

### Electrophoretic Mobility Shift Assay

EMSA were performed using nuclear extracts as follows: 10 ug of nuclear extract in a 10-ul reaction volume was incubated on ice for 40 min. Double-stranded Oligonucleotide DNA probes, (κB: 5'-AGTTGA*GGGGACTTTCCC*AGGC-3', and Oct-1:5'-TGTCGA*ATGCAAAT *CACTAGAA-3') were end-labeled with ^32^P-γ, and applied to a 4% nondenatured polyacrylamide gel. Equal loading of nuclear extracts was confirmed by determining Oct-1 DNA binding activity. After electrophoresis, the gel was dried for 1 h at 80°C and exposed to Kodak X-ray film (Eastman Kodak Co., Rochester, NY, USA) at -80°C.

### Semi- quantitative Reverse Transcription-PCR

Total RNA was extracted from 1 × 10^6 ^of ACC-M and ACC-2 cells using TRIzol (Invitrogen, crop. Carlsbad, CA, USA). Aliquots (1 ug) of RNA were reverse transcribed to cDNA and aliquots (4 ul) of cDNA were used as a template for PCR using a PE9700 RT-PCR system (Applied Biosystems, Singapore) according to the manufacturer's instructions. The primers sets were as follows: VEGF (682 bp), 5'ggc tct aga tcg ggc ctc cga aac cat3' and 5'ggc tct aga gcg cag agt ctc ctc ttc3'; β-actin(434 bp), 5'tgt gcc cat cta cga ggg gta tgc3' and 5'ggt aca tgg tgg tgc cgc cag aca3'. Thermocycling conditions were melting at 95°C for 30 s; anneal at 63.5°C (VEGF 27 cycles) for 45s, or 57°C(β-actin 25 cycles) for 30s; extension at 72°C for 30s. The PCR products were analyzed by electrophoresis in a 2% agarose gel. The relative RNA amount was calculated by the Gene Genius gel imaging system (Syngene, UK). All experiments were carried out three times: each time the reading was taken in triplicate and the average and standard deviations were calculated.

### Immuofluorescence double staining and semi-quantitative confocal laser scanning analysis

Cells were fixed with methanol for 10 minutes at -20°C. After permeated with 0.5% Triton X-100 in PBS for 10 minutes at room temperature, cells were blocked by 2% bovine serum albumin for 30 minutes at 37°C. Then cells were incubated with the primary antibodies overnight at 4°C. Primary antibodies were anti-NF-κB mouse monoclonal antibody (Santa Cruz, CA, USA) used at a dilution of 1:100, and anti-VEGF rabbit polyclonal antibody (Santa CruZ, CA, USA) used at a dilution of 1:150. After washing with TBST, the cells were incubated with secondary antibodies, diluted 1:80 in TBST. Secondary antibodies that were used were CY3-conjugated goat anti-rabbit IgG (Sigma, St. Louis, USA) and fluorescein isothiocyanate (FITC)-conjugated goat anti-mouse IgG (Pierce, Rockford, USA). The cells were then washed three times with PBS. Immunofluorescence microscopy was performed using a Leica TCS-SP2-AOBS-MP confocal microscope (Leica Microsystem, Heidelberg, Germany). According to the recommendation of Nakayama [13], the rate of the nuclear localization of p65 was calculated by the following method: count positive nuclear staining NF-κB from total cells and then calculate the percentage. Each of the five cell slides was counted every ten randomly selected in high-power fields (× 200). The relative protein amount of VEGF was calculated according to the mean intensity of the fluorescence on five cell slides by Leica confocal software 2.61.

### Stable Transfection of ACCs Cells with IκBαM and Control vector

The PCMV-IκBαM vector was provided by Professor Chiao (Anderson Cancer center, Huston, Texa, USA). The CMV-IκBαM vector has mutations (S32, 36A) of the NH2 terminus and a COOH-terminal PEST sequence, which specifically inhibits phosphorylation of IκBα. Then ACC-M and ACC-2 cells (1 × 10^6^) were transfected using 15 ul of lipofectamine reagent (Invitrogen, Grand Island, NY, USA) and 4 ug of PCMV-IκBαM or control PCMV vector according to the manufactures instruction. Cells were selected with standard medium containing 800 ug/ml and 600 ug/mlG418 respectively. Fourteen days later, neo-resistant colonies were isolated by trypsinization and established as subcultures. The cells resistant G418 and stable express exogenous IκBαM were used for subsequent analyses.

### Endothelial cell migration assay

Endothelial cell motility assay was carried out as described previously [14]. Briefly, a 6-well plate was coated with type-I collagen and incubated overnight at 37°C. UVECs were seeded into the coated wells at a density of 2 × 10^6 ^cells/well and incubated for 24 h. Then scrape the monolayer cells to make a clear area with a narrow tip and wash with serum-free medium. 1.0 ml CM from ACC-M and ACC-2 cells were added into the wells and incubated for 24 h, and the 1.0 ml serum free DMEM medium was used as the control. Then the cells were fixed and stained using Acridine Orange and photographed by fluorescence microscope (Leica Microsystem, Heidelberg, Germany).

### Tube formation assay

500 ul type I collagen gel solution (0.3%) containing 5 mg/ml of human fibronectin (Collaborative Research Inc. Lexingtonf, USA), 100 ul 10 × DMEM and 400 ul NaOH-Heppers buffer was mixed in an ice cold condition and pipetted into a 6-well plate and kept for 30 min at 37°C or gelatinization. UVECs were seeded into the layer of the gel at a density of 5 × 10^4 ^cells/well with 10% FBS medium. After 24 h, the medium was replaced by 0.8 ml CM from ACC-M and ACC-2 cells, which was added with 0.2 ml 10% FBS DMEM medium to reach the 2% FBS-CM final concentration. The 1.0 ml 2% FBS medium was used as the control [15]. After 3 days, the tubular structures organized and gradually elongated and formed networks by UVEC cells. Then the cells were fixed and stained using Acridine Orange and photographed by a fluorescence microscope (Leica Microsystem, Heidelberg, Germany).

### In vitro three-dimensional angiogenic assay model

In vitro three-dimensional angiogenic assay model was then performed as described previously [2]. Briefly, the type I collagen gel solution mixture was put on a filter of Millicell-CM inserts and placed in 6-well plates. After gelatinization of the collagen solution, UVEC cells (1 × 10^5 ^cells) were seeded on the surface of the gels in the Millicell and cultured in 10% FBS. When the UVEC cells reached subconfluence about 24 h after seeding, the medium in the outer wells was replaced by 1.5 ml 2.0% FBS-CM and the 1.5 ml 2.0% FBS medium was used as the control. In the Millicell well, 0.5 ml of 2.0% FBS medium was added. Within a week of culture, UVEC cells started to sprout into the gels beneath the confluent monolayer. Then the gels were fixed and stained using Acridine Orange, and the sprouting structures were observed using a Leica LSM 410 confocal laser scanning microscope.

### Statistical evaluation

The experiments were repeated thrice and all data are presented as mean ± S.D. Statistical analysis was performed by ANOVA test. The Spearman rank correlation coefficient test was applied for the correlation among the expression of NF-κB and VEGF. P-values less than 0.05 were considered to be significant.

## Results

### NF-κBp65 activity and mRNA expression level of VEGF in ACCs cell lines

**Figure 1:**
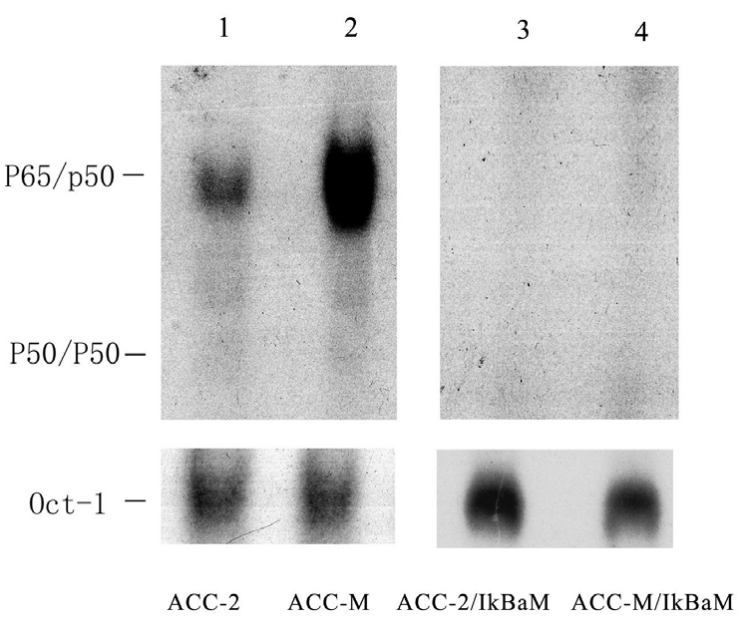
EMSA showed the activity of NF-κB p65 in ACC-M, ACC-M/IκBαM, ACC-2 and ACC-2/IκBαM cell lines. *Arrows *indicate the migration of the induced NF-κB DNA-binding complexes. Migration of the free probe is not shown. The Oct-1 motif was used as a control for quality and quantity of cell extract.

NF-κBp65 DNA binding activity in the nuclear extracts from ACC-M and ACC-2 cell lines was showed in Figure 1, lane 1–2. There was a constitutive NF-κBp65 activity in ACC-M and ACC-2 cell lines. In the ACC-M, the intensity of the shift band of p65/P50 was much stronger than that in ACC-2.

**Figure 2:**
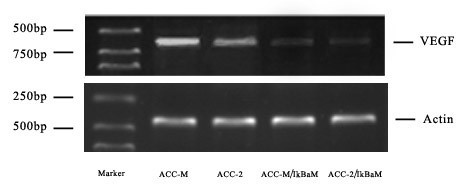
RT-PCR showed the mRNA expression of VEGF in ACC-M, ACC-2, ACC-M/IκBαM and ACC-2/IκBαM cell lines.

Figure 2 showed the mRNA expression band of VEGF in ACC-2 and ACC-M. The mean level of VEGF mRNA in ACC-M (0.575 ± 0.10) was about 2-fold than that in ACC-2 (0.309 ± 0.11), and the statistical difference was deemed significant (*P *< 0.01) [see additional file 1].

### Nuclear localization of NF-κB and semi-quantity immunofluorescence of VEGF in ACCs cell lines

**Figure 3:**
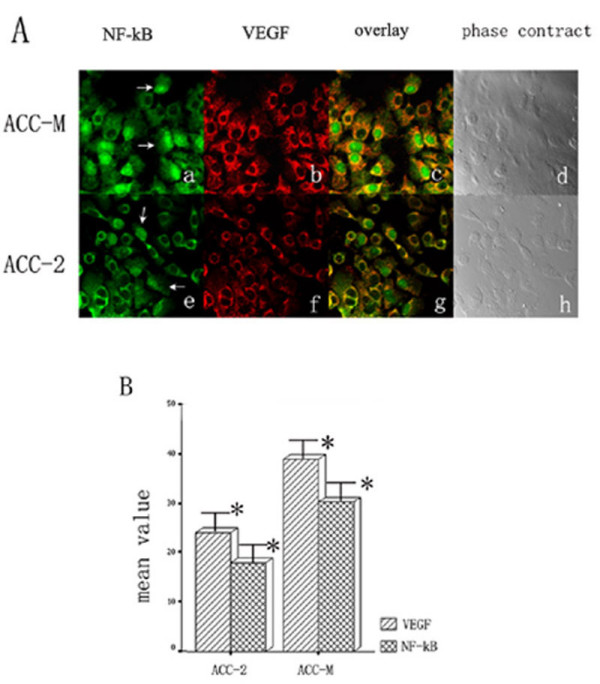
Immuofluorescence double staining and semi-quantitative confocal laser scanning analysis showed NF-κB p65 and VEGF expressed in ACC-M and ACC-2 cell lines. As figure 3A showed, the rate of NF-κB p65 nuclear localization (a) (white arrow) and VEGF staining intensity (b) in ACC-M was higher than that in ACC-2 (e) and (f). As figure 3B shows, bars represent the mean value of immunofluorescence intensity of VEGF and nuclear staining rate of NF-κB p65 in two cell lines, *P *< 0.01(*).

As Figure 3A showed, the green fluorescence is the NF-κB p65 staining, which was detected in almost all of the cytoplasm but only some of the nucleus in ACCs cells. The mean rate of NF-κB p65 nuclear staining detected in ACC-M and ACC-2 was 30.35 ± 2.52% and 17.97 ± 1.50%, respectively. The rate of NF-κB p65 nuclear localization in ACC-M was significantly higher than that in ACC-2 (*P *< 0.01).

The red fluorescence detected in the cytoplasms of ACCs cells is the VEGF staining. The mean immunofluorescence intensities of VEGF in ACC-M and ACC-2 cell lines were 38.98 ± 4.98 and 24.10 ± 1.57, respectively (Figure 3). The expression level of VEGF in ACC-M was significantly higher than that in ACC2 (*P *< 0.01, Figure 3B).

The Spearman Correlation analysis was performed to quantitate the association between two variables. The expression level of VEGF was significantly correlated to NF-kB p65 nuclear localization in both of the ACC-M and ACC-2 cell lines (*P *< 0.01).

### Down regulation of VEGF mRNA expression by inhibition of NF-κBp65 activation

ACC-M and ACC-2 cells were transfected with the mutant IκBα expression vector. The constitutive NF-κBp65 activity found in ACC-M and ACC-2 cell lines was completely abolished in ACC-M/IκBαM and ACC-2/IκBαM cell lines (figure 1, lane 3–4). Furthermore, the constitutive mRNA expression level of VEGF was effectively inhibited by over-expression of IκBαM in ACC-M/IκBαM and ACC-2/IκBαM cells (figure 2, lane 3–4).

### ACCs cell lines enhanced the migration of endothelial cells

**Figure 4:**
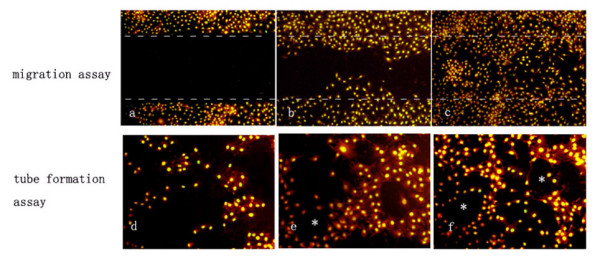
Migration and tube formation of endothelial cells. UVECs were cultured with the conditioned medium from control (a), ACC-2(b) and ACC-M (c) for migration assay. Edges of the denuded areas are marked by dashed lines. UVECs were cultured with the conditioned medium from control (d), ACC-2(e) and ACC-M (f) for tube formation assay. One of tube-like structure was marked by asterisk (*) in figure b and c.

After 24 hours of culture, the scrape-wounded UVECs monolayers incubated with medium from ACC-M showed that cells migrated into the denuded area more densely than the ones incubated with medium from ACC-2 (Figure 4b, c). On the other hand, in the medium from control cultures, the cells sparsely migrated into the denuded area and the wound remained open (Figure 4a).

### ACCs cell lines stimulated the tube formation of endothelial cells

In the presence of medium from ACC-M, UVECs formed organized elongated tube-like structures resembling capillaries with an extensive network (Figure 4f). While in the presence of medium from ACC-2, the formation of the tubular-like structures and networks by UVECs were decreased (Figure 4e). However, cultured with medium from the control, tube formation was diminished and no such well-organized structures were observed (Figure 4d). The results indicated that ACCs cells with high metastasis ability could stimulate the tube formation of angiogenesis.

### ACCs cell lines stimulated the UVEC sprouting into the gels matrix

**Figure 5:**
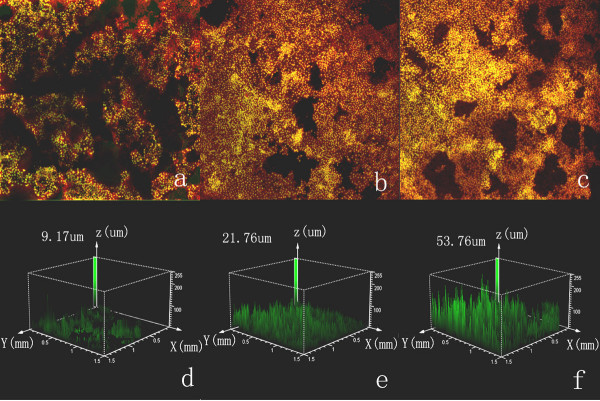
In vitro three-dimensional angiogenic assay of endothelial cells. Control (figure 5a), ACC-2 (figure 5b) and ACC-M (figure 5c) induce UVECs sprouting into the gel matrix (white arrow) and the mean thickness of sprouting was showed by figure 5d, figure 5e and figure 5f. UVECs cultured with ACC-M (figure 5f) shows more cell sprouting points and thicker sprouting layers than cultured with ACC-2 (figure 5e).

After a week of culture with medium from ACC-M in the outer well, the tubules structure grew further thickening into the gel and the monolayer on surface of the gel gradually reach sub-confluence. At the thickened branch points of the tubules structure, the UVECs sprouting into the gel matrix could be observed by the confocal laser scanning microscope (Figure 5c). The mean thickness of sprouting was 53.96 um (Figure 5f). While cultured with the medium from ACC-2, there were less UVECs sprouting into the gel matrix (Figure 5b) and the mean thickness of sprouting was 21.76 um (Figure 5e). It showed that UVECs cultured with ACC-M (figure 5f) presented more cell sprouting points and thicker sprouting layers than cultured with ACC-2 (figure 5e). On the other hand, UVECs cultured with medium from the control only formed uncompleted tubule structures on the surface of gel, and almost no sprouting cells could be observed.

## Discussion

The great potential for hematogenous metastasis at an early stage is one of the unique characters of ACCs. Our previous study showed that the high expressions of angiogenesis related factors NF-κB and VEGF were significantly correlated to lung metastasis and solid histotype, which present high metastasis tendency [3]. In the current study, we used high and low metastasis cell lines of human ACCs-ACC-M and ACC-2- to compare the angiogenic related factor NF-κB and VEGF expression levels. We also compared the ability of angiogenesis between the two cell lines by in vitro cell migration, tube formation and sprouting assay.

The tumor cells induced secretion of angiogenesis factors which is commonly observed in most aggressive tumors [16]. Among various angiogenic factors, the most notable is VEGF, which exerts its mitogenic activity especially on endothelial cells [5]. Our findings revealed that NF-κBp65 activity detected in the high metastasis cell line ACC-M was much greater than that in the low metastasis cell line ACC-2, and the VEGF mRNA expression level in the high metastasis cell line ACC-M was almost 2-fold than that in the low metastasis cell line ACC-2. Moreover, the protein staining intensity of VEGF in ACC-M is also significantly higher than that in ACC-2. The results are consistent with the evidence that the tumor-induced VEGF expression and NF-κB activity correlated with tumor metastasis [8,17]. It was suggested that the aberrant activity of NF-κB and VEGF level might be the possible mechanisms involved in the high metastasis ability of ACC-M cell lines.

In the neovascularization of several neoplasms, research have shown the potent angiogenic factor VEGF, whose genes have a κB binding site, are regulated by activated NF-κB [18,19]. Once triggered and activated, freed NF-κB, in the heterodimer of the p65 and p50 subunits, translocates from the cytoplasm into the nucleus and binds to the specific sequence in the promoter of target genes. In our study, it was found that the constitutive activity of NF-κBp65 detected in ACC-M and ACC-2 cells. In these cell lines, inhibition of NF-κBp65 activity by a PCMV vector mediated expression of phosphorylation defective mutant of IκBα effectively inhibited the expression of VEGF mRNA. Furthermore, the nuclear staining rate of NF-κBp65 was significantly correlated with VEGF protein level in both ACC-M and ACC-2 cell lines. These results suggest that NF-κBp65 is involved in the regulation of VEGF expression. This hypothesis is supported by the findings of others. The study of Tamami et al. [20] demonstrated the inhibitors of the transcription factors NF-κB completely prevented the advanced glycation end products (AGE)-induced up-regulation of VEGF mRNAs and the subsequent increase in DNA synthesis in endothelial cells. The authors suggested that NF-κB activation might be involved in the AGE-elicited angiogenesis through overproduction of auto-secretion VEGF proteins. Huang et al. demonstrated a concordant increase in NF-κB activity with the elevated VEGF mRNA in ovarian cancer cells [21]. In this cell line, stable expression of mutated IκBα resistant to degradation, decreased NF-κB activity and reduced VEGF mRNA expression, suggesting that the regulation of VEGF by NF-κB is mediated at the transcription level.

The angiogenic process includes endothelial cell activation, proliferation, migration, tube formation, and capillary sprouting [22]. It increases the opportunity to improve the development of metastases [23] in many malignant tumors, such as gastro-intestinal tumors [24], colorectal cancer [25], lung adenocarcinoma [26], and hepatocellular carcinoma [27]. VEGF and NF-κB have been reported to be crucial in new blood vessel formation [14]. Those angiogenic-related factors increase the opportunity for malignant cells distance metastases through the leakage basement vessel, and hence profoundly influence the prognosis of cancer patients [5,17]. To further indicate the angiogenesis abilities in different metastasis cells lines of ACCs, the in vitro angiogenesis model was also employed. Through in vitro three-dimensional angiogenic assay model, our present study showed ACC-M, the highly metastasis cell clone, could remarkably enhanced the migration and tube formation of UVECs and induce UVECs sprouting into the gel matrix. ACC-M cells presented higher angiogenic activity might result from its higher VEGF expression levels and NF-κB activity which were crucial in tumor angiogenesis [8,20,21]. The findings of Ishibashi's experiment also showed that specific VEGF antibody could inhibit the in vitro angiogenic activity in human salivary gland carcinoma cells [2]. As a result, it might raise the possibility that the high metastasis cell line ACC-M present higher angiogenic-related factors might facilitate the further angiogenesis.

In conclusion, our current study has shown human ACCs cells with high metastasis potential express high levels of constitutive NF-κB p65 activity and VEGF expression. Suppression of NF-κB p65 activity through stable expression of a phosphorylation defective IκBα mutant (S32, 36A) significantly decreased VEGF expression. Furthermore, ACC-M could remarkably stimulate the migration and tube formation of endothelial cells and induce UVECs sprouting into the gel matrix. The results indicate that in ACCs, cells with higher metastasis potential might present greater angiogenesis ability. Our further study will investigate the function of NF-κB p65 signaling in angiogenesis and metastasis of ACCs cells.

## Competing interests

The author(s) declare that they have no competing interests

## Authors' contributions

**JZ**: Contributions to conception and design, acquisition of data, analysis and interpretation of data. Contributions to draft the manuscript

**BP**: Contributions to conception and design, revise the manuscript for important intellectual content and given final approval of the version to be published.

## Pre-publication history

The pre-publication history for this paper can be accessed here:



## Supplementary Material

Additional file 1 Statistical analysis of the difference of VEGF mRNA level. The mean level of VEGF mRNA in ACC-M was significant higher than that in ACC-2. Click here for file
