# Supplementary figures and images for: In vitro angiogenesis and expression of nuclear factor κB and VEGF in high and low metastasis cell lines of salivary gland Adenoid Cystic Carcinoma
Source: BMC Cancer. 2007 Jun 1;7:95. doi: 10.1186/1471-2407-7-95 (PMC1903362; doi:10.1186/1471-2407-7-95)

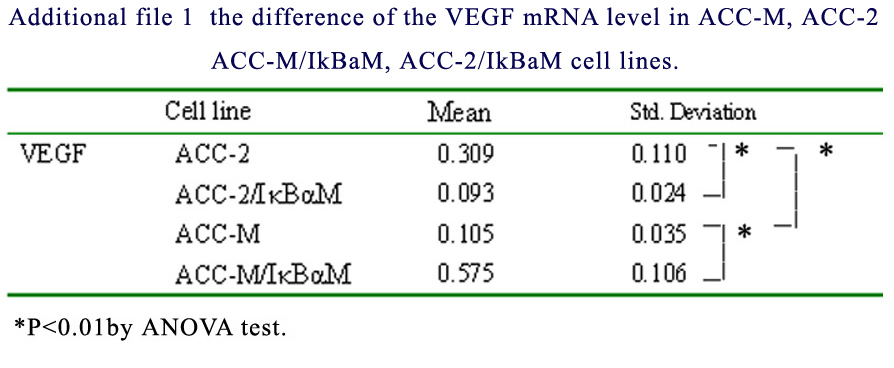

Supplement: Additional file 1 — Statistical analysis of the difference of VEGF mRNA level. The mean level of VEGF mRNA in ACC-M was significant higher than that in ACC-2. [file 1471-2407-7-95-S1.jpeg]
